# About the Influence of PEG Spacers on the Cytotoxicity of Titanate Nanotubes-Docetaxel Nanohybrids against a Prostate Cancer Cell Line

**DOI:** 10.3390/nano11102733

**Published:** 2021-10-15

**Authors:** Alexis Loiseau, Julien Boudon, Céline Mirjolet, Véronique Morgand, Nadine Millot

**Affiliations:** 1 Laboratoire Interdisciplinaire Carnot de Bourgogne, UMR 6303 CNRS Université Bourgogne Franche-Comté, BP 47870, CEDEX, 21078 Dijon, France; alexis_loiseau@yahoo.fr; 2 INSERM 1231, Cadir Team, CEDEX, 21078 Dijon, France; cmirjolet@cgfl.fr; 3 Radiotherapy Department, Georges-Francois Leclerc Cancer Center, CEDEX, 21079 Dijon, France; vmorgand@cgfl.fr

**Keywords:** titanate nanotubes, polyethylene glycol, colloidal stability, docetaxel, cytotoxicity, prostate cancer cells

## Abstract

The association between chemotherapeutic drugs and metal oxide nanoparticles has sparked a rapidly growing interest in cancer nanomedicine. The elaboration of new engineered docetaxel (DTX)-nanocarriers based on titanate nanotubes (TiONts) was reported. The idea was to maintain the drug inside cancer cells and avoid multidrug resistance mechanisms, which often limit drug efficacy by decreasing their intracellular concentrations in tumor cells. HS-PEG_n_-COOH (PEG: polyethylene glycol, n = 3000, 5000, 10,000) was conjugated, in an organic medium by covalent linkages, on TiONts surface. This study aimed to investigate the influence of different PEG derivatives chain lengths on the TiONts colloidal stability, on the PEG_n_ density and conformation, as well as on the DTX biological activity in a prostate cancer model (human PC-3 prostate adenocarcinoma cells). In vitro tests highlighted significant cytotoxicities of the drug after loading DTX on PEG_n_-modified TiONts (TiONts-PEG_n_-DTX). Higher grafting densities for shorter PEGylated chains were most favorable on DTX cytotoxicity by promoting both colloidal stability in biological media and cells internalization. This promising strategy involves a better understanding of nanohybrid engineering, particularly on the PEGylated chain length influence, and can thus become a potent tool in nanomedicine to fight against cancer.

## 1. Introduction

In the last two decades, titanate nanotubes (TiONts) have generated increased attention for their great potential since their discovery by Kasuga et al. [1,2]. Recently, they have been considered with strengthened interest by our group and others for biomedical applications [3]. It includes the dopamine detection [4], DNA transfection [5] and adsorption [6], orthopedics and dental implants [7], bioimaging, when conjugated with superparamagnetic iron oxide nanoparticles (SPIONs) [8], with dithiolated diethylenetriaminepentaacetic acid-modified gold nanoparticles (Au@DTDTPA NPs) [9] or with fluorescent probes (phthalocyanine) [10], safe nanocarrier [5,11,12], drug delivery (genistein and docetaxel) [9,13,14,15] and cancer cell radiosensitization [9,15,16]. TiONts present a needle-shaped morphology with an internal cavity [9,14] and they can be internalized with no cytotoxicity induction and maintained inside cells for at least 10 days in vitro [5,12,16]. Indeed, the tubular nanoparticles (NPs) are more readily internalized than their spherical counterparts of a similar specific surface area [5,17,18]. It has also been shown that the shape and functionalization of NPs, used as carriers, affect the colloidal stability in suspension [14,19,20,21], attachment of selective groups [22,23], biodistribution [24,25,26,27] and interaction mode between nano-objects and cells [19,20,26,28,29]. Hence, the surface chemistry of TiONt-nanocarriers allows via hydroxyl groups further functionalization and complementary functionalities such as stability and biocompatibility in physiological conditions, which are a mandatory requirement for biomedical applications [9,11,14,21,27].

To date, very few studies have investigated the functionalization of TiONts for drug delivery purposes. Multidrug resistance mechanisms often limit drug efficacy by decreasing intracellular concentrations of drugs in tumor cells [30]. Hence, the development of engineered nanocarriers to maintain the drug inside cancer cells and thus improve treatment efficacy seems highly relevant [27,31]. The most used type of stabilization is the steric hindrance which improves the long-term colloidal stability under biological conditions and thus increases the blood circulation time of NPs [19,20,32]. Indeed, this strategy with bulky molecules is much more efficient at higher salt concentrations than the one using electrostatic interactions [33]. Polyethylene glycol (PEG) grafting has become the most widely used approach to provide stealth properties to drug nanocarrier against the reticuloendothelial system. It avoids the adsorption of opsonin proteins due to the neutrality, lipophilicity at the same time as hydrophilicity, and capacity for hydration of the PEG moiety [34,35,36,37]. PEG are also biocompatible and biodegradable polymers used in a wide range of molecular weights (usually from 160 to 20,000 g∙mol^−1^ for nanomedicine) and approved by the Food and Drug Administration (FDA) [32,36]. Besides, it has already been proven to promote the passive targeting of NPs by the enhanced permeability and retention (EPR) effect [38,39] and to improve the therapeutic efficacy [20,40]. PEGylated chain length of the polymer allows a screening effect to be observed [9], which enables the renal clearance of NPs as well as a reduced liver uptake [38]. Several studies reported the impact of grafting density and PEGylated chain length on the biological behavior of different NPs [19,20,35,36], such as liposomes [29,41,42], polymeric [43,44,45] and inorganic NPs (quantum dots, gold NPs, iron oxide NPs, silica NPs, etc.) [20,46,47,48,49,50] as well as carbon nanotubes [51,52]. Nevertheless, no study, to the best of our knowledge, has been conducted on PEG-modified TiONts. It has been established that both higher PEG density and PEGylated chain length onto the nano-object surface allows improving the colloidal stability while reducing surface interaction with its environment (in particular nonspecific adsorption of proteins), hence minimizing its detection by the immune system, as well as their uptake by cells [47,53]. Adsorbed proteins may either facilitate cancer cell entry or mark inorganic NPs for macrophage detection followed by their clearance from the body [47]. For instance, higher grafting densities lead to less protein adsorption and lower NPs uptake by cell lines while shorter PEG chain lengths result in higher cellular uptake in cell lines at the cost of greater nonspecific protein adsorption. Thus, it is necessary to find a compromise between the PEG density and chain length used.

In the present study, the core strategy is based on the use of different PEG spacer (HS-PEG_n_-COOH; n = 3000, 5000, 10,000) between TiONts and one chemotherapeutic agent, herein docetaxel (DTX), to observe the influence of PEGylated length on nanotube properties and promote the interactions of DTX-functionalized TiONts with the tubulins present into microtubules [54,55]. DTX is a clinically well-established cytotoxic drug with inhibitory properties on mitosis [31]. It has been approved by FDA especially for the treatment of hormone-refractory prostate cancers [56,57]. In previous studies, TiONts have been synthesized by a hydrothermal process and functionalized with (3-aminopropyl)triethoxysilane (TiONts-APTES) to provide additional amine functions for PEG_3000_ and DTX conjugation, making them promising drug carriers [9,14,15]. This report describes the functionalization of TiONts-APTES with different PEG_n_ molecular weights in an organic medium to analyze the influence of PEGylated chain lengths on the colloidal stability and PEG_n_ density as well as the cell survival after loading DTX molecules on PEG_n_-modified TiONts (TiONts-PEG_n_-DTX). In vitro efficacy of these latter is evaluated on a human PC-3 prostate adenocarcinoma cells using 3-(4,5-dimethylthiazol-2-yl)-5-(3-carboxymethoxyphenyl)-2-(4-ulfophenyl)-2H-tetrazolium (MTS) assay. The analysis of each elaboration step of TiONts-PEG_n_-DTX is performed using several characterization techniques such as thermogravimetric analysis (TGA), transmission electron microscopy (TEM), ζ-potential measurement, X-ray photoelectron spectroscopy (XPS), Fourier-transformed infrared (FTIR), and UV-visible spectroscopies.

## 2. Materials and Methods

### 2.1. Materials

Titanium dioxide (TiO_2_) rutile was obtained from Tioxide (Calais, France). 1-ethyl-3-(dimethylaminopropyl) carbodiimide hydrochloride (EDC), N-hydroxysuccinimide (NHS), tris (2-carboxyethyl)-phosphine hydrochloride (TCEP), and *p*-maleimidophenyl isocyanate (PMPI) were acquired from Thermo Scientific (Illkirch, France). (3-aminopropyl) triethoxysilane (APTES), sodium hydroxide (NaOH), benzotriazole 1-yl oxytripyrrolidinophosphonium hexafluorophosphate (PyBOP), ethanol, and *N*,*N* diisopropylethylamine (DIEA) were purchased from Sigma Aldrich (Saint Quentin Fallavier, France). Alpha-Thio-Omega-carboxy polyethylene glycol (HS-PEG_3000_-COOH, MW = 3073 g∙mol^−1^; HS-PEG_5000_-COOH, MW = 4847 g∙mol^−1^; HS-PEG_10,000_-COOH, MW = 9515 g∙mol^−1^) were obtained from Iris Biotech GmbH (Marktredwitz, Germany). Docetaxel (DTX) was purchased by BIOTREND Chemikalien GmbH (Cologne, Germany). Phosphate buffered saline (PBS) 1× solution (Acros Organics BVBA, Geel, Belgium), and dimethyl sulfoxide (DMSO extra dry, anhydrous 99.99%) (Acroseal) were acquired from Fisher Chemicals (Illkirch, France). Borate buffered saline (0.1 M; pH 8.5) was prepared from boric acid (99.8%). The ultrafiltration stirred cell (Model 8400, 400 mL) and membranes (regenerated cellulose 100 kDa) were purchased from Merck Millipore (Molsheim, France). Only ultrapure water was used for the preparation of aqueous solutions (*ρ* = 18 MΩ.cm).

### 2.2. Titanate Nanotube (TiONts) Synthesis

Bare TiONts were synthesized by a hydrothermal process in a basic medium, according to ref [9,14]. Briefly, TiO_2_ rutile as a precursor (1 g) was ultrasonicated in a NaOH aqueous solution (10 M, 250 mL) for 30 min at 375 W (Sonics Vibra-Cells, Newton, CT, USA). Then, the mixture was added into a Teflon reactor with mechanical stirring at 120 rpm and heating at 155 °C for 36 h. After cooling to room temperature, TiONts were washed and purified by centrifugation (24,000× *g*, 10 min), dialysis (Cellu·Sep tubular membranes of 12–14 kDa), and ultrafiltration (regenerated cellulose membranes with a molecular weight cut-off (MWCO) of 100 kDa) with ultrapure water.

### 2.3. Amine-Functionalized TiONts (TiONts-APTES) Preparation

Amine-functionalized TiONts (TiONts-APTES) were prepared from a silane-coupling agent, presenting high reactivity with hydroxyl groups on the surface of the material. Subsequently, TiONts were functionalized by APTES via hydrolysis and condensation process in an ethanol/water mixture (50:50 *v:v*) under reflux and magnetic agitation (60 °C, 5 h) [9,14,58]. The molar ratio between hydroxyl functions of TiONts and APTES was 1:3. After the synthesis, the suspension was ultrafiltered (100 kDa) and freeze-dried.

### 2.4. Functionalization of TiONts-APTES by Polyethylene Glycol with Different Ethylene Oxide Chain Lengths (PEG_3000/5000/10,000_)

The same method was used for the grafting of different heterobifunctional polymers (HS-PEG_3000_-COOH: PEG_3000_; HS-PEG_5000_-COOH: PEG_5000_; HS-PEG_10,000_-COOH: PEG_10,000_) onto the TiONts-APTES surface. First, carboxyl groups of heterobifunctional polyethylene glycols have been activated with PyBOP (molar ratio was 1:1) in DMSO in the presence of DIEA (in 6 × excess) under nitrogen flow and magnetic stirring for 30 min. Then, TiONts-APTES were dispersed in DMSO before adding in the activation solution, for 24 h under magnetic agitation and nitrogen flow. Polymers were attached to the amine groups of APTES with a molar ratio of 1:1. Finally, the products (TiONts-PEG_n_: TiONts-PEG_3000_, TiONts-PEG_5000_, and TiONts-PEG_10,000_) were washed by centrifugation (20,000× *g*, 20 min, mainly to remove DMSO less compatible with the ultrafiltration membrane), then purified by ultrafiltration (500 kDa) with ultrapure water.

### 2.5. Modification and Grafting of Docetaxel on TiONts-PEG_n_

The modification and grafting of the therapeutic agent (docetaxel, DTX) were described by Loiseau et al. [9,14] and occurred in the same conditions for each TiONts-PEG_n_. Briefly, DTX and the PMPI crosslinker were dissolved in DMSO (the molar ratio of DTX to PMPI was 1:4) and then added in borate buffered saline (0.1 M; pH 8.5) under magnetic agitation at 25 °C for 24 h. The resulting PMPI-activated DTX (DTX-PMPI) solution was then dialyzed (0.5–1 kDa) to remove unreacted PMPI and lyophilized to obtain a yellowish powder. TiONts-PEG_n_-DTX were synthesized from TiONts-PEG_n_ and DTX-PMPI (large excess) using TCEP for cleavage of disulfides in PBS (0.1 M; pH 7.4). The mixture was homogenized beforehand in an ultrasonic bath and placed under magnetic stirring at 25 °C for 24 h. TiONts-PEG_n_-DTX were washed and purified by dialysis and ultrafiltration (500 kDa), and then, freeze-dried.

### 2.6. Surface Area Measurements

Specific surface area measurements were performed using a Micromeritics Tristar II apparatus (Micromeritics Instrument Corp., Norcross, GA, USA). Samples were outgassed in situ under 20 mTorr pressure for 16 h at 100 °C. The specific surface area value (S_BET_) from N_2_ gas adsorption was calculated from Brunauer–Emmett–Teller (BET) method.

### 2.7. Thermogravimetric Analysis (TGA)

TGA (TA instrument, Discovery TGA, Newcastle, UK) was used to determine the amount of the molecules on the surface of the TiONts after each grafting step. All powders were analyzed with a temperature ramp of 10 °C∙min^−1^ from 50 to 800 °C under an airflow rate of 25 mL∙min^−1^. The experiments were reproduced from 2 to 10 times for each sample.

### 2.8. ζ-Potential Measurements

Zeta potentials of nanoparticle suspensions were measured with a Malvern Nano ZS instrument (Worcestershire, UK) supplied by DTS Nano V7.11 software (Worcestershire, UK). pH titrations from 3 to 11 were carried out using aqueous solutions of HCl (0.1 M), NaOH (0.1 M), or NaOH (0.01 M). Before each measurement, the powder was dispersed in an aqueous NaCl solution (10^−2^ M) and sonicated for 10 min.

### 2.9. UV-Visible Absorbance Measurements

UV-visible absorbances at 600 nm were measured using Shimadzu UV-2550 UV-visible spectrophotometer (Tokyo, Japan). Turbidimetric studies of nanoparticle suspensions were made in PBS (0.1 M; pH 7.4) at 25 °C (one measurement/5 min).

### 2.10. X-ray Photoelectron Spectroscopy (XPS)

A PHI 5000 Versaprobe apparatus (ULVAC-PHI, Osaka, Japan) from a monochromatic Al Kα1 X-ray source (EKα1 (Al) = 1486.7 eV with a 200 μm diameter spot size, an accelerating voltage of 12 kV, and a power of 200 W) was used to record XPS measurements. Powders were pressed on an indium sheet before analysis. Data analysis and curve fittings were realized with CasaXPS processing, and MultiPak software (ver. 9.0.1, Osaka, Japan) was employed for quantitative analysis. A Shirley background was subtracted and Gauss (70%)–Lorentz (30%) profiles were applied. The charge effects were minimized by a neutralization process and the Ti_2p_ peak at 458.7 eV was used as a reference to correct the charge effects. The resolution was 2.0 eV for global spectra and 1.3 eV for windows corresponding to selected lines.

### 2.11. Transmission Electron Microscopy (TEM)

Nanotube morphology and agglomeration state characterization were performed using a JEOL JEM-2100F (Tokyo, Japan), with an accelerating voltage of 200 kV and fitted with an ultra-high pole-piece achieving a point-to-point resolution of 0.19 nm. Samples were prepared by evaporating a diluted suspension of nanoparticles onto the carbon-coated copper grids.

### 2.12. Fourier Transformed Infrared (FTIR) Spectroscopy

A Bruker Vertex 70v (Billerica, MA, USA) supplied by OPUS version 3.1 software (Billerica, MA, USA) was used to record FTIR spectra using the KBr method. The pellets were made by mixing 2 mg of the sample within 198 mg of dried KBr.

### 2.13. Inductively Coupled Plasma (ICP) Spectroscopy

Determination of titanium content in nanohybrids in contact with cells was performed by ICP coupled to mass spectrometry (ICP-MS) analysis (ThermoScientific iCAP 6000 series ICP Spectrometer (Waltham, MA, USA)). A dried sample of centrifuged cells in contact with nanohybrids (TiONts-PEG_3000_-DTX or TiONts-PEG_10,000_-DTX) were dissolved in 1 mL aqua regia during 72 h in a PTFE reactor submitted to microwaves. The resulting solutions were diluted to a total volume of 5 mL with 2% HNO_3_ before analysis at the Ti wavelength (336.121 nm). The measured concentration (in ppt) allowed the calculation of the Ti concentration (in µg∙L^−1^) and deducing the mass of nanohybrids per cell (knowing the number of cells per sample).

### 2.14. Cell Culture of Human PC-3 Prostate Adenocarcinoma

Human PC-3 prostate adenocarcinoma cells (ATCC, Manassas, VA, USA) were cultured in Dulbecco’s modified Eagle medium (DMEM) with 10% fetal calf serum (Deutscher, France) at 37 °C, 5% CO_2_, and 95% humidity.

### 2.15. In Vitro Evaluation of Nanohybrid Cytotoxicity

Androgen-independent PC-3 prostate cancer cells were seeded in 96-well plates at a concentration of 3000 cells/well to determine the DTX cytotoxicity on the nanohybrid surface. Cells were incubated at 37 °C in 190 μL of drug-free culture medium (DMEM), with 10% fetal calf serum for 24 h before treatment (when the cells were at around 20% confluence). Cytotoxicity tests were performed with five samples at each concentration of free DTX (positive control), DTX-PMPI, TiONts-PEG_3000_-DTX, TiONts-PEG_5000_-DTX, and TiONts-PEG_10,000_-DTX. PC-3 cells were then incubated (+ 10 μL of drug in 190 μL of culture medium) with a range of equivalent DTX concentrations from 0.5 to 500 nM (100 nM of DTX corresponds to 0.18 μg, 0.23 μg, and 2 μg of TiONts-PEG_3000_-DTX, TiONts-PEG_5000_-DTX, and TiONts-PEG_10,000_-DTX per well from TGA, i.e., nanohybrid concentrations of 0.9 μg∙mL^−1^, 1.15 μg∙mL^−1^ and 10 μg∙mL^−1^, respectively). After 96 h of incubation corresponding approximately to 4 cycles, cell viability was evaluated using MTS assay (Promega Corporation, Madison, WI, USA) according to Mirjolet et al. [9,14,15,16]. Results were expressed as relative absorption at 490 nm relative to the untreated control.

## 3. Results and Discussion

**Figure 1:**
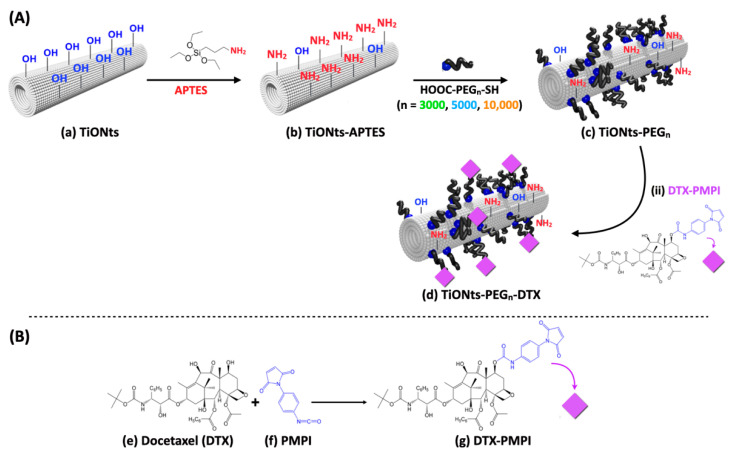
Illustration of (**A**) TiONts (**a**) and step-by-step pre-functionalization with APTES (**b**) and α-acid-ω-thiol-polyethylene glycol (**c**) using one of the three different carbon chain lengths (PEG_n_; n = 3000, 5000, or 10,000); (**B**) in the second step, DTX (**e**) and PMPI (**f**) were combined to form PMPI-modified DTX (**g**) to create the final nanohybrid TiONts-PEG_n_-DTX (**d**).

The titanate-based nanohybrid has been elaborated step-by-step following the strategy described in Figure 1 to create a very attractive nanocarrier for chemotherapy.

**Figure 2:**
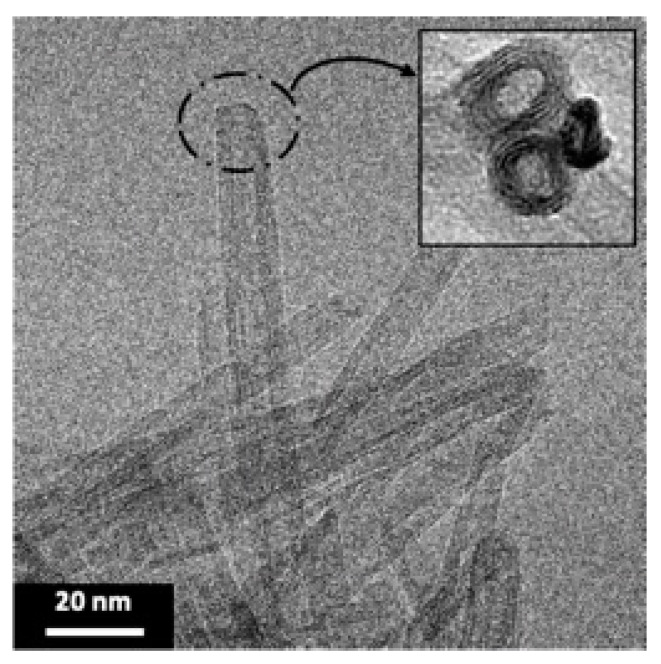
TEM micrograph shows the elongated morphology of TiONts with a coiled spiral-shaped structure and an internal cavity.

Transmission electron microscopy (TEM) is used to highlight the formation of TiONts with a coiled spiral-shaped structure and an internal cavity, as presented in Figure 2 and described in previous work [12,14,59]. They are 10 ± 1 nm in outer diameter, 4 ± 1 nm in inner diameter, as well as 170 ± 50 nm in length and they, present a high specific surface area due to their specific morphology (S_BET_ = 174 ± 1 m^2^∙g^−1^).

**Figure 3:**
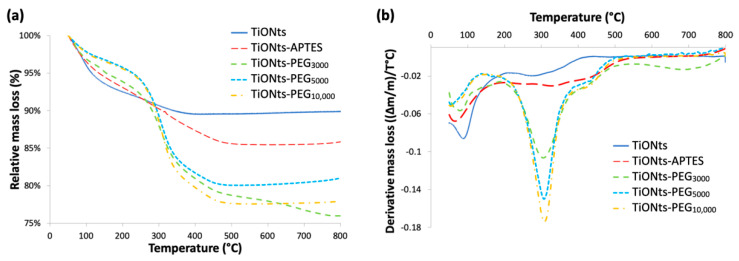
(**a**) TGA and (**b**) derivative curves of bare TiONts and functionalized-TiONts under air atmosphere.

TGA results have first demonstrated the effective synthesis of the TiONts-PEG_n_ nanohybrids (Figure 3; Table 1). TGA analysis shows a more consequent mass loss after PEG_n_ grafting due to the additional organic matter on TiONts as is the case at each successive step of grafting (details on the calculation are given in Figure S1). Moreover, as expected, the greater the molecular weight (MW) of polymer, the more important the mass losses in TGA but, on the contrary, the lower the grafting yields (0.09 PEG_3000_∙nm^−2^; 0.05 PEG_5000_∙nm^−2^; 0.03 PEG_10,000_∙nm^−2^). The PEG density of each polymer grafted onto the TiONts surface seems to be related to the polymer chain length (the longer the chains, the lower the PEG density), in particular for reasons of steric hindrance: the anchoring group might be embedded into the organic layer limiting thus the ease of access for grafting. PEGylation density is commonly related to the Flory radius (RF) of the grafted PEG, the distance between grafted PEG (D =$\;\sqrt{4S_{{\mathrm{PEG}}_{n}}/\pi }$), or the length/thickness of the grafted PEG layer. There are two main conformations that PEG chains can acquire based on these parameters: “mushroom” (RF < D) or “brush” (RF > D) conformation [36,60,61,62,63]. The determined coverage rates ($S_{{\mathrm{PEG}}_{n}}$) obtained by the DMSO/PyBOP pathway correspond to areas of 11, 20, and 33 nm^2^ relative to PEG_3000_, PEG_5000_, and PEG_10,000_, respectively. These values are approximately 5 to 8-fold lower than the theoretical projected surface areas (πRF^2^) for polymer chains of 3, 5, and 10 kg∙mol^−1^, for which the values reported in the literature of RF are about 4.4, 6.0, and 9.1 nm [63,64], respectively, which corresponds to a covering surface of 61, 113 and 260 nm^2^. Moreover, the calculated distances (D) between grafted PEG_n_ are 3.7, 5.0, and 6.5 nm relative to PEG_3000_, PEG_5000_, and PEG_10,000_. These results then indicate a PEG_n_ brush conformation (RF > D), relatively sparse, less for the PEG_10,000_ chains grafted which seem more stretched than those of PEG_3000_ and PEG_5000_ (RF/D = 1.2, 1.2 and 1.4 for PEG_3000_, PEG_5000_, and PEG_10,000_, respectively). Considering these ratios close to 1, the conformation of the PEG coatings would be more likely between little stretched chains and loose mushrooms.

**Figure 4:**
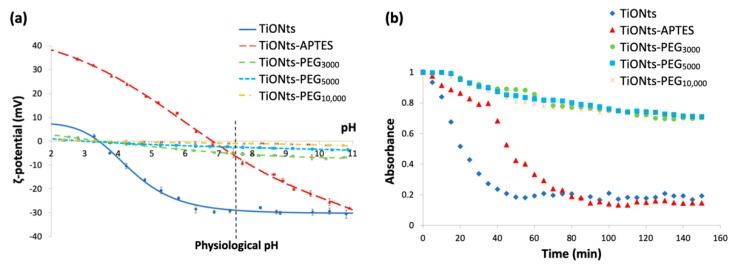
(**a**) ζ-potential curves as a function of pH in NaCl (10^−2^ M) of bare TiONts and different functionalized-TiONts (the vertical dashed line corresponds to the physiological pH). (**b**) Turbidimetric studies: colloidal stability of functionalized-TiONts suspensions (PBS 0.1 M; pH 7.4) over 150 min following their absorbance at 600 nm as a function of time.

As shown in Figure 4a, *ζ*-potential measurements highlight the presence of PEG_n_ on the surface of APTES-modified TiONts with the observation of a significant charge shielding for the different PEGylated chain lengths. This effect is clearly pronounced when the PEGylated chain length increases and even more in this synthesis pathway leading to higher PEG grafting yields when compared to the water pathway published earlier [14]. These measurements confirm TGA results by proving a PEG brush conformation as the carbon chain length tends to hide the charges present on the TiONts surface. ζ-potential is then close to 0 mV over the entire studied pH range. Thus, ζ-potential for TiONts-PEG_n_ indicates an isoelectric point (IEP) at pH 3.5 and varies between 0 mV and −5 mV at physiological pH (pH 7.4) by suggesting that the steric effect mainly governed the colloidal stability at this pH value.

**Figure 5:**
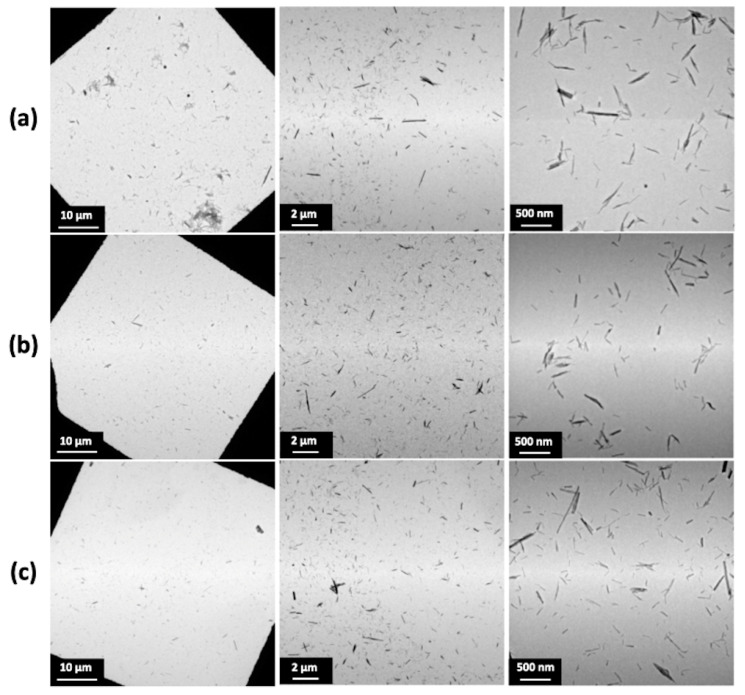
TEM images of TiONts-PEG_n_ dispersion state: (**a**) TiONts-PEG_3000_, (**b**) TiONts-PEG_5000_, and (**c**) TiONts-PEG_10,000_.

Turbidimetric analyses in Figure 4b highlight the colloidal stability of the different PEG_n_-functionalized TiONts’ suspensions under physiological conditions (PBS 0.1 M; pH 7.4), which is correlated with TEM images Figure 5. The absorbance measurements as a function of time (150 min; *λ* = 600 nm) demonstrate better colloidal stability for TiONts-PEG_n_ suspensions in PBS (0.1 M; pH 7.4) than bare TiONts or TiONts-APTES. Nevertheless, the colloidal stability remains substantially the same whatever the molecular weight of PEG.

Figure 5 shows the dispersion of TiONts-PEG_n_ in the same suspension concentration. The PEG_n_ grafting on the nanotube surface greatly enhances nanohybrid individualization, regardless of the molecular weight used, as compared with TiONts and TiONts-APTES (Figure S2). Despite the different grafting densities of PEG_n_ on TiONts, the PEGylated chain length offsets the number of grafted molecules and leads to a similar dispersion state of the nanohybrids. Namely, a decrease in the PEG density does not lead to a greater agglomeration of nanotubes. Thus, this excellent dispersion state highlights the effectiveness of the peptidic coupling with PyBOP on the grafting yields and the colloidal stability of nanotubes functionalized by PEG chains in a brush conformation.

XPS analyses have been carried out to evaluate the chemical composition on the surface of the different nanohybrids (Table 2). The XPS quantitative analysis reveals the presence of PEG_n_ on nanotubes, with a significant increase in carbon and oxygen rates compared to TiONts-APTES and as the PEG MW is greater. In addition, XPS shows that sulfur (originally at the end of PEG chains and involved in the C–S coupling between PEG and DTX-PMPI) is only observed for both TiONts-PEG_3000_ (0.3%) and TiONts-PEG_5000_ (0.2%) samples. This could be explained by a thinner layer of lower MW PEG (3000 and 5000) on the TiONts’ surface even if their grafting density is higher compared to greater PEG MW in TiONts-PEG_10,000_ samples grafted in smaller amounts (Table 1). This is correlated by a significant decrease in the Na_KLL_ component as the corresponding chemical element, belonging to the chemical composition of TiONts, becomes more hidden when using a polymer with increasing molecular weight, until complete disappearance (Table 2). This is probably related to either the depth of XPS analysis (a few nanometers) as the grafting of PEG_n_ partially hides the titanate core or the repeated washings (ultrafiltration after each grafting step) which decrease the Na content below the detection limit. By comparison with the literature, it is interesting to note that the grafting of PEG_3000_ on TiONts using a PyBOP crosslinker rather than EDC/NHS carbodiimide coupling [14] leads to higher concentrations of carbon and oxygen in the resulting TiONts-PEG_3000_ nanohybrid: C/Ti = 1.5 and O/Ti = 3.5 for PyBOP versus C/Ti = 1.0 and O/Ti = 2.5 for EDC/NHS. Besides, from the TGA study, the grafting ratio of PEG_3000_ on TiONts-APTES is almost doubled with the use of PyBOP versus carbodiimide coupling (0.09 PEG_3000_∙nm^−2^, as shown in Table 1, versus 0.05 PEG_3000_∙nm^−2^, respectively) and the improved grafting yield confirms that the PEG_n_ grafting is more efficient in the organic medium than in water.

**Figure 6:**
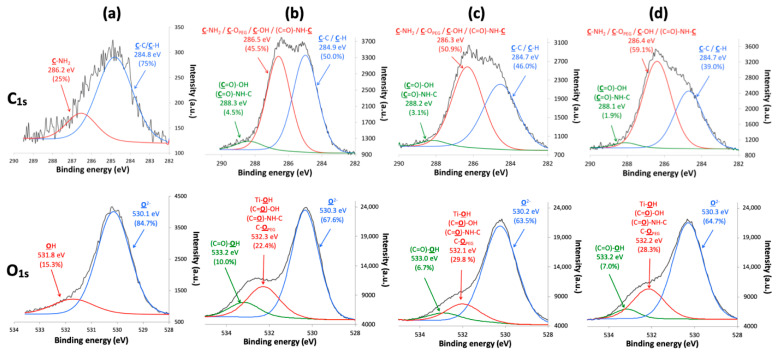
XPS spectra with the fitted peaks of C_1s_ and O_1s_ for (**a**) TiONts-APTES, (**b**) TiONts-PEG_3000_, (**c**) TiONts-PEG_5000_, and (**d**) TiONts-PEG_10,000_.

The decomposition of the C_1s_ and O_1s_ threshold of the TiONts-PEG_n_ samples also highlights the formation of the characteristic bonds of the PEG_n_ grafting on TiONts (Figure 6). The C_1s_ peak of the different TiONts-PEG_n_ shows one component located at 286.4 eV (C–O_PEG_, C–OH and (C=O)–NH–C) and the appearance of a new component at 288.2 eV ((C=O)–OH and (C=O)–NH–C) attributed to the formation of secondary amide bonds characterizing the PEG_n_ grafting on TiONts-APTES and the polymers proper bonds. The appearance of a new component in the O_1s_ peak attributed to the carboxyl function of polymers ((C=O)–OH; 533.2 eV) and the slight shift of the component located at 532.2 eV, in comparison with TiONts-APTES, show the presence of new characteristic grafting bonds. This contribution of the latter corresponds to (C=O)–OH, (C=O)–NH–C, and C–O_PEG_, proving once again the presence of polymers as well as the formation of the covalent amide bonds. The proportions of components for the C_1s_ and O_1s_ thresholds vary according to the carbon chain length of PEG_n_ (from n = 3000 to 10,000). Indeed, a decrease is observed for the components located at 530.3 eV (O^2−^, from 67.6% to 64.7%) and 533.2 eV ((C=O)–OH, from 10% to 7%) on the O_1s_ threshold and at 288.2 eV ((C=O)–OH; (C=O)–NH–C) for the C_1s_ peak (4.5%, 3.1%, and 1.9%, respectively, for n = 3000 to 10,000). This observation demonstrates not only a shielding effect when the size of the PEG carbon chain increase by hiding in-depth bonds but also a decrease of the grafted PEG amount due to a higher steric hindrance ((C=O)–OH; (C=O)–NH–C).

**Figure 7:**
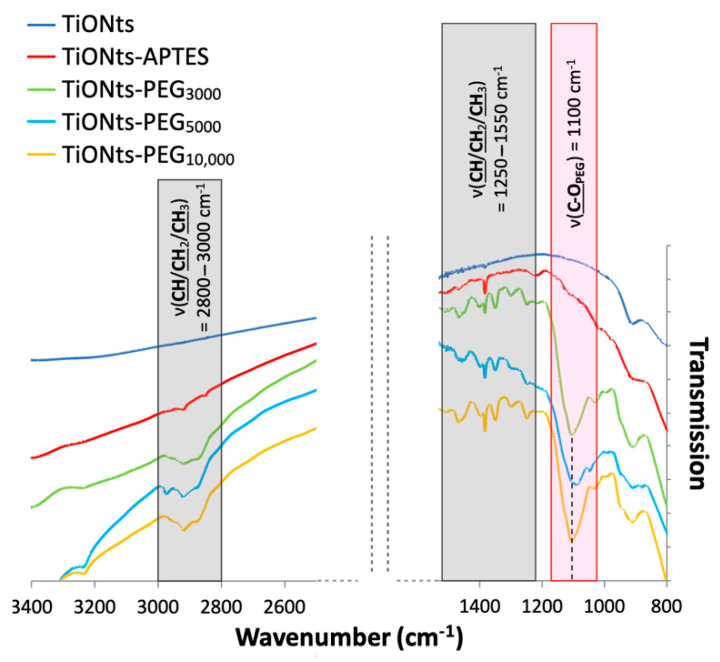
FTIR spectra of TiONts, TiONts-APTES, TiONts-PEG_3000_, TiONts-PEG_5000_ and TiONts-PEG_10,000_ between 3400–2500 cm^−1^ and 1600–800 cm^−1^.

The FTIR spectra correlate with the TGA and XPS results by confirming the PEG_n_ presence on TiONts surface, notably with a new C-O_PEG_ specific band to the ethylene glycol repeat units (Figure 7). Indeed, the most intense vibration bands, attributed to C-O_PEG_ at 1100 cm^−1^ (red highlight on FTIR spectra) and aliphatic carbons (CH/CH_2_/CH_3_) between 3000–2800 cm^−1^ and 1550–1250 cm^−1^ (dark gray highlight on FTIR spectra), can be assigned to the characteristic bonds of PEG. Once more the PEG_n_ grafting thanks to DMSO/PyBOP showed more intense vibration bands by comparison with the EDC/NHS coupling in our previous study [14].

DTX pristine molecule is not readily reactive to be attached as is to TiONt nanohybrids. To activate the coupling of DTX, *p*-maleimidophenyl isocyanate (PMPI) crosslinkers have been reacted thanks to their isocyanate groups on one of the hydroxyl groups of DTX. In a second step, the maleimide groups of PMPI crosslinkers have been used to form new carbon-sulfur bonds with the end thiols of PEGs to eventually form TiONts-PEG_n_ preferentially at pH 7.4 in PBS. Whereas the reaction is possible with both thiol and amine functions above pH 7.5, the specificity of maleimide for thiols is preserved when the reaction is carried out between pH 6.5 and 7.5 [65] (Figure S3).

**Figure 8:**
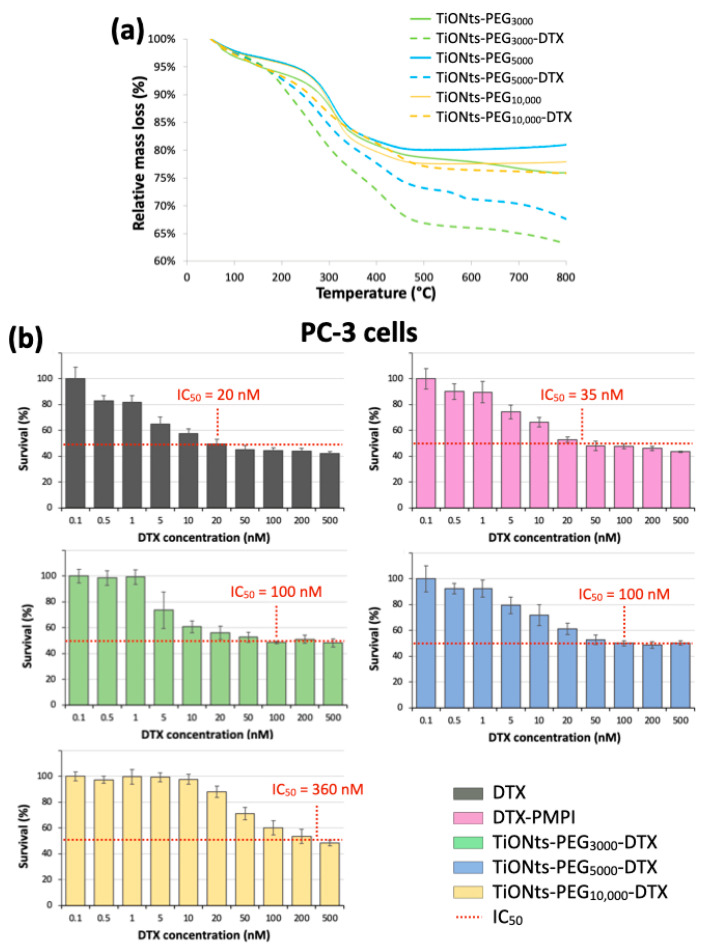
(**a**) TGA curves of TiONts-PEG_n_ and TiONts-PEG_n_-DTX under air atmosphere. (**b**) Survival curves (MTS cytotoxicity assays) on PC-3 cell lines after incubation of DTX, DTX-PMPI, and TiONts-PEG_n_-DTX (mean ± SD) (the horizontal dotted lines allow for an estimate of the different nanohybrids’ IC_50_). The studied range was from 0.5 to 500 nM in DTX concentration, which corresponded to a concentration range of 4.5 × 10^−3^ to 4.5 μg∙mL^−1^, 5.75 × 10^−3^ to 5.75 μg∙mL^−1^, and 5 × 10^−2^ to 50 μg∙mL^−1^ for TiONts-PEG_3000_-DTX, TiONts-PEG_5000_-DTX and TiONts-PEG_10,000_-DTX, respectively.

The grafting yields of DTX-PMPI on the surface of TiONts-PEG_n_ were determined by TGA and are presented in Table 1 and Figure 8a. The amount of grafted therapeutic molecules, in the case of TiONts-PEG_3000_ prepared thanks to DMSO/PyBOP, is higher than the one obtained, in a previous study, by EDC/NHS coupling in water [14]: 0.32 DTX-PMPI∙nm^−2^ versus 0.24 DTX-PMPI∙nm^-2^, respectively. Indeed, the number of grafted polymers on the TiONts surface is also higher, promoted by our new strategy of TiONts-DTX nanohybrid elaboration. Thus, the smaller the PEGylated chain size, the greater the DTX-PMPI amount. Furthermore, the number of DTX on TiONts-PEG_3000_ and TiONts-PEG_5000_ have been found greater than that of sulfur groups initially present at the nanotube surface: 0.32 DTX-PMPI∙nm^−2^ versus 0.09 PEG_3000_∙nm^−2^ for TiONts-PEG_3000_-DTX and 0.24 DTX-PMPI∙nm^−2^ versus 0.05 PEG_5000_∙nm^−2^ for TiONts-PEG_5000_-DTX. To some extent, DTX-PMPI seems to have another strong interaction with the surface (in addition to the one with PEG_n_ thiols). Additional experiments are then carried out and confirm the presence of modified-DTX on simple contact with the use of an inactive polymer grafted onto TiONts-APTES, preventing any chemical reaction with PMPI-DTX (Figure S4). As its grafting ratio does not vary despite repeated purifications, these DTX-PMPI molecules being thus less likely embedded within PEG chains. DTX-PMPI molecules could presumably interact with the remaining amine groups of APTES (not already functionalized by PEG_n_), or could also be trapped inside the cavity of nanotubes. Indeed for DTX-PMPI grafting, the reaction carried out at pH 7.4 used to promote the formation of covalent bonds with the thiol groups of PEG_n_, might have undergone a pH drift to higher values resulting in unexpected coupling with the amine function of APTES [65], as described above. It should be noted that the grafting ratio of DTX-PMPI on TiONts-PEG_10,000_ corresponds to the same as that of PEG_10,000_ (0.03 DTX-PMPI∙nm^−2^ and 0.03 PEG_10,000_∙nm^−2^ for TiONts-PEG_10,000_-DTX). This observation seems to be a direct consequence of the total shielding of APTES by the PEG_10,000_ layer. Thus, DTX-PMPI tends to attach mainly to the thiol groups provided by the PEG_10,000_, contrary to PEG_3000_ and PEG_5000_, which would also have another way of adhesion, improving the DTX grafting yields and probably, the therapeutic effect of nanohybrids on tumor cells.

Additionally, ICP-MS measurements have been realized on cells incubated with TiONts-PEG_n_-DTX, the results of which are presented in Table 3. First, a dose-effect can be noticed with an increase in the mass of nanohybrids per cell when the DTX concentration is doubled (10 to 20 nM). However, this effect is more pronounced when the PEG chain is shorter: when DTX concentration varies from 10 to 20 nM, the TiONts-PEG_3000_-DTX mass is increased 5-fold compared to the 2.5-fold increase in the case of TiONts-PEG_10,000_-DTX nanohybrids. This may be due to the steric hindrance of the polymer shell, bigger in the case of MW = 10,000 g∙mol^−1^. The thinner shell could thus improve interaction with cells.

MTS assays on a PC-3 human prostate cancer cell lines evaluate the cytotoxicity of DTX (modified or not) and associated nanohybrids (Figure 8b). The first results reveal that TiONts-PEG_n_-DTX nanohybrids have a cytotoxic activity according to their half-maximum inhibitory concentration (IC_50_). DTX-nanohybrids cytotoxicity are lower than that of DTX (IC_50_: 20 nM; black curve) and DTX-PMPI (IC_50_: 35 nM; pink curve), as described in a previous report [9,14,15]. Note that PMPI-activated DTX molecules have a slightly lower cytotoxic activity compared with free DTX, most probably because of the covalent bond formed between PMPI and DTX. Nevertheless, the survival curves show also a “plateau effect” at high doses around the IC_50_ for each TiONts-PEG_n_-DTX nanohybrid and suggest a phenomenon called mitotic catastrophe, which is a type of cell death, as already observed in the literature [66,67,68]. Indeed, DTX binding can inhibit mitotic cell division by blocking the microtubule repolymerization and prevent further cell proliferation, but some cells could continue to replicate their DNA without dividing [69]. This leads to a higher survival rate for high doses in DTX due to hypermetabolic cells before inducing the apoptosis or necrosis shortly after mitosis dysfunction. Even with this phenomenon, the cytotoxic activity of TiONts-PEG_3000_-DTX (green curve) is more than three times greater than that observed for the same nanohybrids elaborated in water (IC_50_: 100 nM versus 360 nM in ref [14], respectively) and is close to that of a previous study by using the same organic way (IC_50_: 82 nM) [9]. PEG brush conformation may affect the biological performance of these nanohybrids, as already demonstrated with PEG-modified carbon nanotubes [70], being most favorable on DTX cytotoxicity while promoting both colloidal stability in biological media and the interaction of the therapeutic agent with microtubules. In addition, the IC_50_ of TiONts-PEG_10,000_-DTX (IC_50_: 360 nM; yellow curve) is greater than the IC_50_ of TiONts-PEG_3000_-DTX and TiONts-PEG_5000_-DTX (IC_50_: 100 nM; green and blue curves, respectively). Therefore, the PEGylated chain length plays an important role in the DTX interactions with the PC-3 prostate cancer cells and, as corroborated by the ICP-MS results, these results show that DTX has a better efficiency when attached on shorter-chains (PEG_3000/5000_ versus PEG_10,000_) which tends indeed to promote the interaction between DTX and microtubules. Despite a similar surface charge and dispersion state for all TiONts-PEG_n_-DTX nanohybrids, a lower internalization rate for TiONts-PEG_10,000_-DTX can occur in comparison with TiONts-PEG_3000/5000_-DTX. Thus, about the PEGylated chain, the higher the grafting densities, the shorter the lengths, the greater the cytotoxicity. These achievements are then very promising to understand the influence of the PEGylated chain length in nanomedicine to improve tumor cell internalization and might ensure better access of DTX to microtubules to fight prostate cancer.

## 4. Conclusions

TiONts were successfully functionalized to develop docetaxel-grafted nanohybrids using a step-by-step process. Each grafting step was characterized by different techniques to prove their success. The grafting of PEG_n_ has been achieved via a peptide coupling with PyBOP in DMSO on the surface of TiONts-APTES and the resulting TiONts-PEG_n_ nanohybrids have significantly improved the colloidal stability of suspensions under physiological conditions consistent with the targeted biomedical applications. The high number of polymers on the nanotube surface (0.09 PEG_3000_∙nm^−2^; 0.05 PEG_5000_∙nm^−2^; 0.03 PEG_10,000_∙nm^−2^) allowed to significantly improve the amount of therapeutic agent (DTX) modified by PMPI on nanohybrids, especially with the use of smaller PEGylated chain lengths (PEG_3000_: 0.32 DTX∙nm^−2^; PEG_5000_: 0.24 DTX∙nm^−2^; PEG_10,000_: 0.03 DTX∙nm^−2^). Indeed, the steric hindrance generated by PEG on nanotubes was progressively improved with increasing molecular weight. In vitro (MTS) tests highlighted satisfactory cytotoxicities of DTX for each TiONts-PEG_n_-DTX nanohybrids on human PC-3 prostate adenocarcinoma cells. Finally, this study demonstrated that the PEG grafting with high molecular weights on TiONts (in our case: PEG_10,000_) decreased the cytotoxic activity of DTX and reduce the internalization in cells. Thus, higher grafting densities of shorter PEG chain lengths have been found more favorable to greater cytotoxicities. Understanding the influence of PEGylated chain lengths allows one to move towards an approach to fight tumor cells and in this context, titanate nanotubes appear as new theranostic tools to improve the efficacy of cancer treatment.

## Figures and Tables

**Table 1:** Detailed analysis of relative mass loss and graft ratio of TiONts and modified-TiONts.

Sample	Initial Temperature of Degradation (°C)	Relative Mass Loss (%)	Degraded Molecular Weight (g∙mol^−1^)	Molecule∙nm^−2^ (Average)	Number of Loaded Molecules Per TiONt (*)
TiONts	190	2.6	18	10.2 (±1.5) OH	-
TiONts-APTES	175	6.3	58	2.6 (±0.2) NH_2_	14,230
TiONts-PEG_3000_	170	17.6	3073	0.090 (±0.005) PEG_3000_	490
TiONts-PEG_5000_	170	16.3	4847	0.050 (±0.003) PEG_5000_	270
TiONts-PEG_10,000_	170	18.8	9515	0.030 (±0.002) PEG_10,000_	160
TiONts-PEG_3000_-DTX	150	29.4	1049	0.32 (±0.02) DTX-PMPI	1750
TiONts-PEG_5000_-DTX	150	25.3	1049	0.24 (± 0.02) DTX-PMPI	1310
TiONts-PEG_10,000_-DTX	150	19.9	1049	0.030 (±0.002) DTX-PMPI	160

(*) A geometrical calculation considering only the external surface of TiONts was used to estimate the number of loaded molecules per TiONts.

**Table 2:** XPS ratio of chemical elements of bare TiONts, TiONts-APTES, TiONts-PEG_3000_, TiONts-PEG_5000_, and TiONts-PEG_10,000_.

Atomic Concentration (%)	C_1s_	O_1s_	Na_KLL_	Ti_2p_	N_1s_	Si_2p_	S_2p_
TiONts	7.3	58.7	13.5	20.5	-	-	-
Elements (TiONts)/Ti	0.3	2.9	0.7	1.0	-	-	-
TiONts-APTES	11.2	56.8	5.7	21.5	2.3	2.5	-
Elements (TiONts-APTES)/Ti	0.5	2.6	0.3	1.0	0.1	0.1	-
TiONts-PEG_3000_	24.1	55.5	-	15.9	2.3	1.9	0.3
Elements (TiONts-PEG_3000_)/Ti	1.5	3.5	-	1.0	0.2	0.1	0.02
TiONts-PEG_5000_	25.1	55.2	-	15.2	2.2	2.1	0.2
Elements (TiONts-PEG_5000_)/Ti	1.6	3.6	-	1.0	0.2	0.2	0.01
TiONts-PEG_10,000_	26.0	55.7	-	14.5	2.3	1.5	-
Elements (TiONts-PEG_10,000_)/Ti	1.8	3.8	-	1.0	0.2	0.1	-

**Table 3:** Masses of PEGylated TiONt-based DTX nanohybrids were calculated from ICP-MS analyses and according to the PEG length (MW = 3000 or 10,000) and the DTX concentration (10 nM or 20 nM).

Sample	Control	Nanohybrid Name and Its Corresponding Docetaxel Concentration			
		**TiONts-PEG_3000_-DTX**		**TiONts-PEG_10,000_-DTX**	
		**10 nM**	**20 nM**	**10 nM**	**20 nM**
Nanohybrid Mass Per Cells (µg/10^6^ Cells)	1.1	64	335	72	179

## Data Availability

The data presented in this study are available on request from the authors.

## Supplementary Materials

The following are available online at https://www.mdpi.com/article/10.3390/nano11102733/s1, Figure S1: (a) TGA curves of bare TiONts under air atmosphere and theoretical calculation of the hydroxyl rates. (b) Theoretical calculation for the graft ratios of functionalized-TiONts; Figure S2: TEM images show the evolution of the dispersion (a) before and (b) after APTES grafting on TiONts surface; Figure S3: (a) Maleimide reacts specifically with a thiol function at pH < 7.5 and (b) lose its specificity to react either with a thiol function or with amine function at pH > 7.5; Figure S4: (a) Polymer (Boc-NH-PEG_3000_-COOH; M = 3173 g∙mol^−1^) having an inactive function (Boc) and carboxyl function to react with an amine group via peptide coupling and (b) TGA curves showing the adsorption of DTX-PMPI upon contact between TiONts-PEG_3000_-Boc and DTX-PMPI (TiONts-DTX were washed by dialysis and ultrafiltration (100 kDa)). (c) Results of relative mass loss and graft ratio of bare TiONts, TiONts-APTES, TiONts-PEG_3000_-Boc and after mixing DTX-PMPI with TiONts-PEG_3000_-Boc.
