# Supplementary material for: About the Influence of PEG Spacers on the Cytotoxicity of Titanate Nanotubes-Docetaxel Nanohybrids against a Prostate Cancer Cell Line
Source: Nanomaterials (Basel). 2021 Oct 15;11(10):2733. doi: 10.3390/nano11102733 (PMC8539671; doi:10.3390/nano11102733)
Supplement: Supplementary file 1 [file nanomaterials-11-02733-s001.zip › nanomaterials-1410516-supplementary-conversion.pdf]

nanomaterials

MDPI

Supporting Information

About the Influence of PEG Spacers on the Cytotoxicity of Titanate Nanotubes-Docetaxel Nanohybrids against a Prostate Cancer Cell Line

Alexis Loiseau [1], Julien Boudon [1,*], Céline Mirjolet [2,3], Véronique Morgand [3] and Nadine Millot [1,*]

[1] Laboratoire Interdisciplinaire Carnot de Bourgogne, UMR 6303 CNRS Université Bourgogne Franche-Comté, BP 47870, CEDEX, 21078 Dijon, France; alexis_loiseau@yahoo.fr
[2] INSERM 1231, Cadir Team, CEDEX, 21078 Dijon, France; cmirjolet@cgfl.fr
[3] Radiotherapy Department, Georges-Francois Leclerc Cancer Center, CEDEX, 21079 Dijon, France; vmorgand@cgfl.fr
* Correspondence: Julien.Boudon@u-bourgogne.fr (J.B.); Nadine.Millot@u-bourgogne.fr (N.M.)

(a)

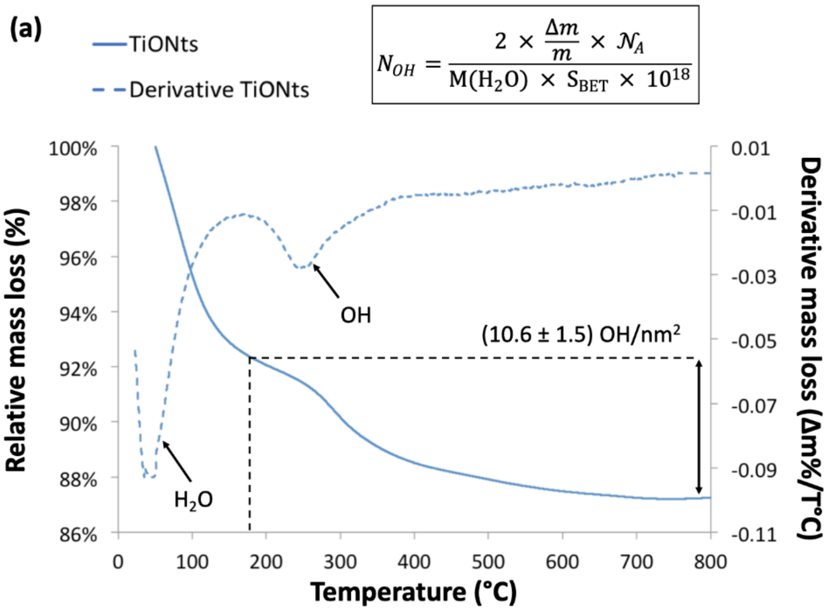

$$N_{OH} = \frac{2 \times \frac{\Delta m}{m} \times \mathcal{N}_A}{\mathrm{M(H_2O)} \times \mathrm{S_{BET}} \times 10^{18}}$$

(b)

$$N_m = N_m = \frac{\left(\frac{\Delta m_2}{m_2} - \frac{\Delta m_1}{m_1}\right) \times \mathcal{N}_A}{\mathrm{M}(m_2) \times \mathrm{S_{BET}} \times 10^{18}}$$

Figure S1. (**a**) TGA curves of bare TiONts under air atmosphere and theoretical calculation of the hydroxyl rates. (**b**) Theoretical calculation for the graft ratios of functionalized-TiONts.

The ratio for the hydroxyl group at the surface of bare TiONts is calculated from the equation presented in this graph, knowing that the condensation of one water molecule involves two hydroxyls. This mass loss is correlated with chemisorbed water using the mass derivative curve between 190 and 800 °C, while the first loss is mainly due to physisorbed water. N_{OH} is the is the number of the hydroxyl groups per nm^2, $\Delta m/m$ is the relative mass loss of chemisorbed water determined by derivative curve (Δm is the mass loss of chemisorbed water (g) and m is the initial mass (g) of the sample TiONts), $\mathcal{N}_A$ is Avogadro's constant, M_{H_2O} is the molecular weight of water ($g \cdot mol^{-1}$), S_{BET} is the specific surface area ($m^2 \cdot g^{-1}$) and 10^{18} is a factor to obtain a ratio per nm^2.

Mass losses increased as additional organic moieties were added to TiONts at each successive step of grafting. N_m is the number of grafted m molecules per nm^2. $\left(\frac{\Delta m_2}{m_2} - \frac{\Delta m_1}{m_1}\right)$ is the difference in relative mass loss between two samples due to grafted organic matter; Δm, which is the mass loss of considered molecule (g), is determined from the second inflection (initial temperature degradation) to 800 °C in derivative curves (Figure 3) (the first inflection is mainly due to physisorbed water) and m is the initial mass (g) of the considered sample. $\mathcal{N}_A$ is Avogadro's constant, $M(m_2)$ is the molecular weight of the degraded molecule ($g \cdot mol^{-1}$), S_{BET} is the specific surface area ($m^2 \cdot g^{-1}$) and 10^{18} is a factor to obtain a ratio per nm^2.

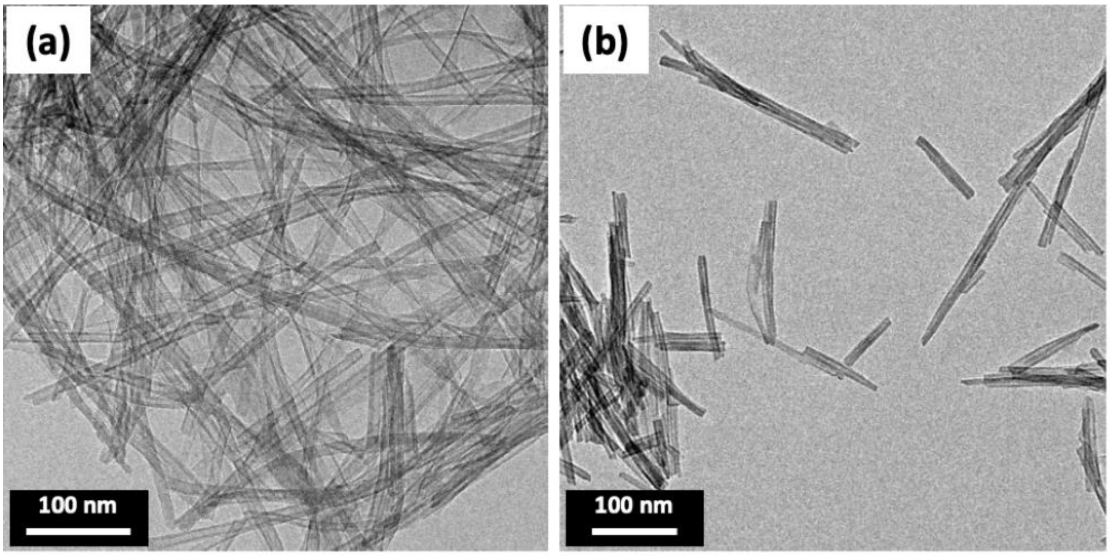

Figure S2. TEM images show the evolution of the dispersion (**a**) before and (**b**) after APTES grafting on TiONts surface.

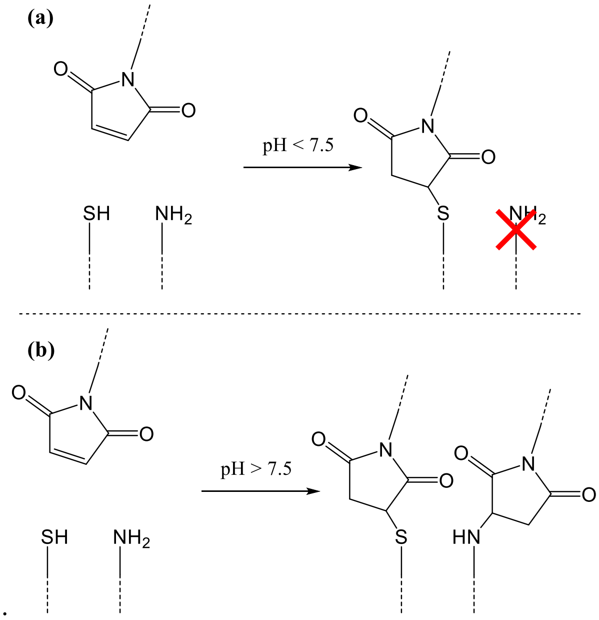

Figure S3. (**a**) Maleimide reacts specifically with a thiol function at pH < 7.5 and (**b**) lose its specificity to react either with a thiol function or with amine function at pH > 7.5.

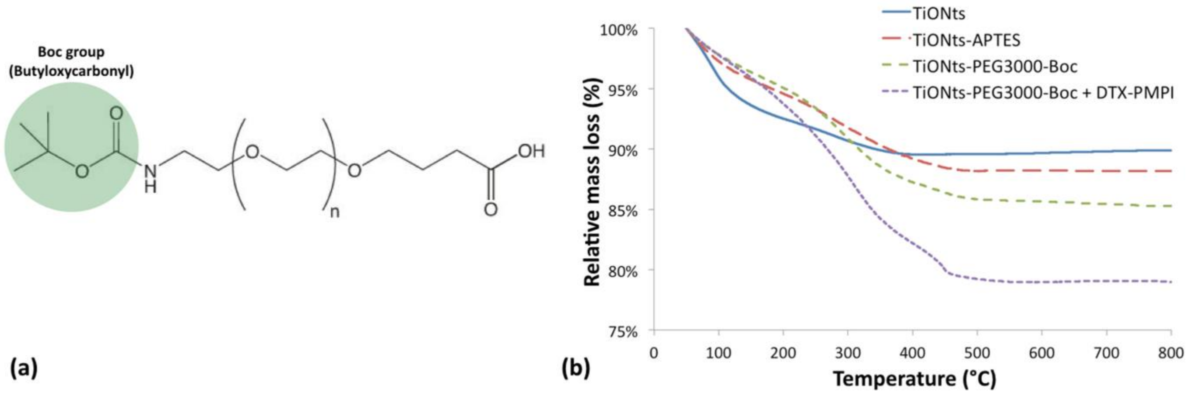

(c)

Sample	Initial temperature of degradation (°C)	Relative mass loss (%)	Degraded molecular weight (g.mol^{-1})	Molecule·nm^{-2} (average)	Number of grafted molecules per TiONt (*)
TiONts	190	2.6	18	10.6 (± 1.5) OH	-
TiONts-APTES	175	6.3	58	5.3 (± 0.5) NH_2	14,230
TiONts-PEG_{3000}-Boc	165	13.2	3173	0.060 (± 0.003) PEG_{3000}-Boc	330
TiONts-PEG_{3000}-Boc + DTX-PMPI	150	17.9	1022	0.13 (± 0.01) DTX-PMPI	710

(*) The number of grafted molecules per TiONt was estimated by means of geometrical calculation considering only the external surface of TiONts.

Figure S4. (**a**) Polymer (Boc-NH-PEG_{3000}-COOH; M = 3173 g·mol^{-1}) having an inactive function (Boc) and carboxyl function to react with an amine group *via* peptide coupling and (**b**) TGA curves showing the adsorption of DTX-PMPI upon contact between TiONts-PEG_{3000}-Boc and DTX-PMPI (TiONts-DTX were washed by dialysis and ultrafiltration (100 kDa)). (**c**) Results of relative mass loss and graft ratio of bare TiONts, TiONts-APTES, TiONts-PEG_{3000}-Boc and after mixing DTX-PMPI with TiONts-PEG_{3000}-Boc.

TGA curves showed the presence of modified-DTX on direct contact with an inactive polymer grafted onto TiONts-APTES bearing a Boc function (the presence of which prevents any chemical reaction with DTX; PEG MW = 3000 g·mol^{-1}, ref. PEG1073), instead of a thiol group. This result demonstrated that there was another interaction mode between DTX-PMPI and TiONt-PEG_n in our study despite repeated purifications. Consequently, it cannot be excluded that DTX-PMPI clung/adsorbed to amine groups of APTES no functionalized by PEG_n, could be in the cavity of nanotubes and/or be trapped within PEGylated chains.
